# The evaluation of the plasma levels of interleukin 17A, thymic stromal lymphopoietin, interferon gamma, tumor necrosis factor-alpha and interleukins IL-2, IL-6, IL-23, and IL-31 in atopic dermatitis patients with dupilumab treatment

**DOI:** 10.3389/fimmu.2026.1748929

**Published:** 2026-02-23

**Authors:** Jarmila Čelakovská, Eva Čermáková, Petra Boudkova, Ctirad Andrýs

**Affiliations:** 1 Department of Dermatology and Venereology Faculty Hospital and Medical Faculty of Charles University, Hradec Králové, Czechia; 2 Department of Medical Biophysics, Medical Faculty of Charles University, Hradec Králové, Czechia; 3 Department of Clinical Immunology and Allergy, Faculty Hospital and Medical Faculty of Charles University, Hradec Králové, Czechia

**Keywords:** atopic dermatitis, cytokines, dupilumab, inflammation and autoimmunity, TSLP

## Abstract

**Background:**

Interleukin -17 (IL-17), particularly IL-17A, thymic stromal lymphopoietin (TSLP), interferon gamma (IFN-γ), tumor necrosis factor-alpha (TNF-α), IL-2, IL-6, IL-23, and IL-31 play a significant role in the pathogenesis of various chronic inflammatory and autoimmune skin diseases.

**Method:**

We conducted an assessment of plasma levels of interleukins IL-17A, TSLP, IFN-γ TNF-α, IL-2, IL-6, IL-23, and IL-31 in 89 atopic dermatitis (AD) patients and in 34 healthy individuals as a control group. The group of AD patients consisted of 27 patients treated with dupilumab for moderate and severe form (15 men, 12 women, mean age of 44.8 years) and 62 AD patients suffering from moderate and severe form without any systemic treatment (35 women,27 men, mean age of 46.3 years). The control group consisted of 34 healthy subjects (22 men, 12 women, mean age of 43.3 years). For screening analysis of plasma levels of cytokines the performance assay Human cytokine Luminex was used. Blood samples were unstimulated and stimulated with phorbol myristate acetate and ionomycin. The levels of IL-17A, TSLP, IFN-γ, TNF-α, IL-2, IL-6, IL-23, and IL-31 were compared in AD patients with the results in control group. Nonparametric Kruskal-Wallis analysis of variance with *post-hoc* Dunn’s test with Bonferroni modification of significance level was used for statistical analysis.

**Results:**

Under unstimulated conditions we found these significant differences:1) Higher IL-17A and TNF-α in dupilumab-treated AD patients vs. healthy controls, suggesting residual Th17 and pro-inflammatory activity despite Th2 blockade. 2) TSLP elevated in both groups of AD patients indicating persistent epithelial barrier stress. 3) Low IFN-γ in both groups of AD patients is consistent with Th2 dominance. Under stimulated conditions we found these significant differences: 1) Lower IL-23 in both groups of AD patients vs. healthy controls suggesting possible impaired Th17 axis activation. 2) Lower IL-2 in patients without systemic treatment vs. healthy controls indicating reduced T-cell activation capacity without biologic therapy. 3) Higher IL-6 in both groups of AD patients vs. healthy controls reflecting ongoing innate/inflammatory activation under both conditions.

**Conclusion:**

Dupilumab effectively suppresses Th2 signaling but does not fully normalize immune balance**;** residual Th17 and innate activity persists. Elevated TSLP and IL-6 suggest that epithelial stress and innate immune activation remain key drivers. Reduced IL-23 and IL-2 under stimulation indicate altered adaptive immune responsiveness in AD patients.

## Highlights

Our study confirmes residual immune activation under dupilumab. Despite effective Th2 blockade, patients show elevated IL-17A and TNF-α at baseline, indicating persistent Th17 and pro-inflammatory activity.
Significantly higher TSLP in all AD patients suggests ongoing epithelial barrier stress independent of biologic therapy.
Our study confirmes altered immune responsiveness under stimulation. AD patients exhibit lower IL-23 and IL-2 compared to healthy controls, pointing to impaired adaptive immune activation, while IL-6 is consistently elevated, reflecting innate inflammatory drive.
We confirmed that immune imbalance persists under biologic therapy. Our findings support a shift toward Th1/Th17 and innate pathways, highlighting the complexity of immune regulation in AD and potential mechanisms for residual symptoms or seasonal flares.

## Introduction

Interleukins and related cytokines—including interleukin-17 (IL-17A), thymic stromal lymphopoietin (TSLP), interferon gamma (IFN-γ), tumor necrosis factor-alpha (TNF-α), IL-2, IL-6, IL-23, and IL-31-play critical roles in the pathogenesis of chronic inflammatory and autoimmune skin diseases.

IL-17A is a pro-inflammatory cytokine implicated in several dermatologic disorders, including psoriasis, atopic dermatitis (AD), acne, alopecia areata, and autoimmune bullous diseases (1–5). It is primarily produced by Th17 cells but is also secreted by CD8^+^ T cells, γδ T cells, and natural killer (NK) cells. IL-17A promotes inflammation through neutrophil recruitment and induction of pro-inflammatory cytokines such as IL-1β and TNF-α (6–11). Although essential for host defense, dysregulated IL-17A production contributes to chronic inflammation and autoimmunity. Its role in oncogenesis remains controversial, with evidence supporting both tumor-promoting and tumor-suppressive effects. In AD, IL-17A expression is generally low due to the Th2-dominant immune profile. Dupilumab, a monoclonal antibody targeting IL-4 and IL-13 signaling, does not directly inhibit IL-17A (7, 10). However, certain AD endotypes-such as intrinsic or Asian phenotypes-exhibit higher IL-17A expression (10). Emerging evidence suggests that dupilumab-induced immune modulation may unmask Th17 activity, potentially leading to increased IL-17A levels (10). Data on plasma IL-17A dynamics during dupilumab therapy remain limited, with most studies focusing on tissue expression or clinical manifestations such as psoriasis-like eruptions (10, 11).

TSLP is an epithelial-derived cytokine that plays a central role in initiating type 2 immune responses, particularly in AD. It activates dendritic cells and promotes Th2 cell differentiation (12). TSLP is highly expressed in lesional AD skin; however, studies examining serum TSLP levels have yielded inconsistent results (13–15). Although dupilumab may indirectly reduce TSLP expression by inhibiting downstream IL-4 and IL-13 signaling, its precise effect on TSLP remains unclear. In certain patients, persistent TSLP expression or dominance of alternative pathways, such as IL-22, suggests that TSLP is not a primary driver in all AD phenotypes (12–24).

IFN-γ, a key Th1 cytokine, plays a complex role in AD. In the acute phase of AD, Th2-driven inflammation suppresses IFN-γ expression, whereas chronic lesions often exhibit increased IFN-γ levels, reflecting a shift toward Th1 immunity (25, 26). IFN-γ can counterbalance Th2 responses but may also impair skin barrier integrity. Distinct AD subtypes show variable IFN-γ expression, with higher levels commonly observed in intrinsic AD (25, 26). Dupilumab therapy may enhance IFN-γ production at the cellular level; however, circulating IFN-γ concentrations often decrease, likely due to broader immune regulation and reduced inflammatory burden (25–30).

TNF-α is a pleiotropic cytokine involved in both the propagation and regulation of inflammation in AD. It contributes to allergic skin inflammation while also supporting immune homeostasis through signaling via TNF receptors TNFR1 and TNFR2, particularly on regulatory immune cells. TNF-α receptor expression correlates with disease severity in AD. Although dupilumab does not directly target TNF-α, studies suggest that it may indirectly reduce TNF-α levels by restoring immune balance (27, 31, 32).

IL-2 is a pivotal cytokine regulating T-cell proliferation and the maintenance of regulatory T cells (Tregs), which are essential for immune tolerance. In AD, IL-2 levels are frequently reduced, reflecting impaired Treg function and sustained immune activation (33). This deficiency may exacerbate inflammation and contribute to disease chronicity. Activated CD4^+^ T cells constitute the principal source of IL-2 (33). IL-2 remains of considerable therapeutic interest: low-dose IL-2 has shown promise in re-establishing immune tolerance in autoimmunity, graft-versus-host disease, and inflammatory disorders, while higher doses are utilized to enhance antitumor immune responses (33).

IL-6 is a pleiotropic cytokine with both pro-inflammatory and immunomodulatory properties (34, 35). It is produced by keratinocytes, fibroblasts, and immune cells in response to barrier disruption and microbial exposure. In AD, IL-6 concentrations are elevated and contribute to inflammation, pruritus, and epidermal barrier dysfunction (34, 35). Additionally, IL-6 suppresses IL-2 signaling, thereby reinforcing Th2-skewed immune responses (34, 35). Although IL-6 is not directly targeted by current AD therapies, it represents a secondary inflammatory pathway that may perpetuate disease activity (34, 35).

IL-23 is a critical regulator of the Th17 axis, promoting IL-17 production and sustaining chronic inflammation (36, 37). While IL-23 is a key pathogenic driver in psoriasis, its role in AD is less pronounced due to the predominance of Th2 immunity (36, 37). Nonetheless, in chronic or mixed-endotype AD, IL-23 may contribute to inflammation and barrier impairment. Reported IL-23 expression in AD is variable, and therapeutic strategies targeting this pathway have demonstrated limited efficacy compared with their success in psoriasis (36, 37).

IL-31 is a neuroimmune cytokine centrally involved in the pathogenesis of pruritus, one of the most debilitating symptoms of AD (38, 39). It is produced by Th2 cells, mast cells, eosinophils, and keratinocytes, and signals through IL-31 receptor A (IL-31RA) expressed on sensory neurons, immune cells, and cutaneous structures (38, 39). IL-31 induces itch by promoting neuronal sensitization and nerve growth, while also contributing to inflammation and barrier dysfunction (38, 39). Elevated IL-31 levels correlate strongly with disease severity and pruritus intensity. Targeted inhibition of the IL-31 pathway, exemplified by the IL-31RA antagonist nemolizumab, has demonstrated significant efficacy in reducing pruritus and improving quality of life in patients with AD (38, 39).

The objective of our study was to assess plasma levels of IL-17A, TSLP, IFN-γ, TNF-α, IL-2, IL-6, IL-23, and IL-31 in AD patients both with and without dupilumab therapy, compared to a healthy control group, during out of pollen season.

The cytokines selected for analysis in this study were chosen to reflect immune pathways that are not directly targeted by dupilumab but are increasingly implicated in immune rebalancing, residual inflammation, and paradoxical clinical phenomena observed during IL-4/IL-13 blockade. Together, these cytokines capture complementary aspects of epithelial activation, adaptive immune polarization, and innate inflammation, allowing a broader assessment of systemic immune reprogramming during long-term dupilumab treatment beyond Th2 suppression alone.

## Materials and methods

Complete dermatological and allergological examination was performed in all patients included in the study. All these patients were examined in the Department of Dermatology, Faculty Hospital Hradec Králové, Charles University, Czech republic.

### Patients

Inclusion criteria were:

1. Age 14 years or older.
2. Diagnosis of AD according to Hanifin and Rajka.

The study included patients with moderate to severe AD who were either untreated with dupilumab or had been receiving dupilumab therapy for at least 24 months.

Exclusion criteria included pregnancy, breastfeeding, and the use of systemic treatments such as cyclosporine, systemic corticosteroids, or other biologic therapies.

AD severity was assessed using the Eczema Area and Severity Index (EASI) and the Scoring Atopic Dermatitis (SCORAD) system. Additionally, patients completed the Patient-Oriented Eczema Measure (POEM) and the Dermatology Life Quality Index (DLQI) to evaluate subjective symptoms and quality of life. These assessments were conducted every three months. In our previous research, we analyzed levels of interleukins IL-4, IL-5, IL-10, IL-13, and IL-33 in the same patient cohort (40).

### Laboratory examination

To analyze plasma levels of IL-17A, TSLP, IFN-γ, TNF-α, IL-2, IL-6, IL-23, and IL-31 we employed the Human Cytokine Luminex assay (Human Luminex Discovery Assay (No.LXSAHM-05).

Blood samples were collected under both unstimulated conditions and following stimulation with phorbol myristate acetate (PMA) and ionomycin. This stimulation method provides a non-specific activation of both innate and adaptive immune cells, enhancing cytokine production.

IL-17A, TSLP, IFN-γ, TNF-α, IL-2, IL-6, IL-23, and IL-31 levels in AD patients were compared to those in the healthy control group. The same laboratory method for the evaluation of interleukins IL-4, IL-5, IL-10, IL-13 and IL-33 was used in our previous study (40).

### Statistical analysis

For statistical analysis, we used the nonparametric Kruskal-Wallis test followed by Dunn’s *post-hoc* test, applying Bonferroni correction to adjust the significance threshold. The null hypothesis assumed no difference among groups, tested against the alternative that at least two groups differ significantly. All statistical evaluations were performed using NCSS 2021 Statistical Software.

## Results

We evaluated two groups of AD patients. The first group included 62 individuals (27 men and 35 women) with moderate to severe AD who were not receiving any systemic therapy. The second group consisted of 27 patients (15 men and 12 women) who had been treated with dupilumab for at least 24 months. The control group comprised 34 healthy blood donors with no signs of allergy and negative total IgE levels. This group was matched to the AD patients in terms of age and gender distribution.

Before initiating dupilumab therapy, the severity of AD was comparable between the two patient groups. Those receiving dupilumab had previously experienced moderate to severe AD, but showed significant clinical improvement after starting treatment, now presenting with mild disease. Dupilumab was administered subcutaneously at a dose of 300 mg every two weeks, alongside topical treatments for skin hydration. Patients not receiving dupilumab were managed with emollients, topical immunomodulators, and corticosteroids with antiseptics during acute flare-ups.

The characteristic of AD patients is recorded in **Table 1** (including SCORAD, EASI, POEM, DLQI, treatment). In **Table 2A** we show the basic characteristics for IL-17A, TSLP, IFN-γ, TNF-α, IL-2, IL-6, IL-23, and IL-31 (both unstimulated and stimulated plasma levels) in patients with and without dupilumab therapy and in control group. Given the rejected normality, we report the median (50th percentile) and the interquartile range (25th, 75th percentile) as a measure of variability.

**Table 1:** Characteristic of atopic dermatitis patients.

Patient characteristics	Dupilumab untreated patients	Dupilumab treated patients	
Age	mean age of 46.3 years (24.2- 52.3)	mean age of 44.8 years (31.6-48.3)	
Number of patients	62 (27 men, 35 women)	27 (15 men, 12 women)	
SCORAD	32.5 (26.5-38.7)	34.1 (30.5-45.2)	Before dupilumab therapy
		9.5 (7.1-18.2)	Average value after 1.5 years of treatment with dupilumab
EASI	30.3 (26.8-38.5)	33.1 (30.1-44.2)	Before dupilumab therapy
		9.1 (8.2-17.2)	Average value after 1.5 years of treatment with dupilumab
POEM	13.9 (10-18)	14.3 (12–21)	Before dupilumab therapy
		4.1 (2 -6)	Average value after 1.5 years of treatment with dupilumab
DLQI	12.9 (9 – 16)	15.5 (11-20)	Before dupilumab therapy
		3.4 (1-5)	Average value after 1.5 years of treatment with dupilumab
Previous systemic treatment	62 patients (100%) - antihistamines - no other previous systematic treatment	27 patients (100%) - antihistamines - cyclosporin	
Previous local treatment	local corticosteroid therapy with antiseptics, emollients, topical immunomodulators	local corticosteroid therapy with antiseptics, emollients, topical immunomodulators	

Control group - 34 healthy subjects (22 men, 12 women), age 43.3 years s.d. 9.5 years.

Explanation: The average values (minimal, maximal values) of SCORAD, EASI, POEM, DLQI are recorded. SCORAD – Scoring of atopic dermatitis, EASI -Eczema Area and Severity Index, POEM, Patient Oriented Eczema Measure; DLQI, Dermatology Life Quality Index.

**Table 2A:** Basic descriptive characteristics for interleukins 17A, TSLP, IFN-γ, TNF-α, IL-2, IL-6, IL-23, and IL-31 (in picogram per milliliter) in patients with and without dupilumab therapy.

Interleukin	Dupilumab yes 27 patients			Dupilumab no 62 patients			Control group 34 healthy subjects		
	Percentile			Percentile			Percentile		
	50th	25th	75th	50th	25th	75th	50th	25th	75th
IL 17A unstimulated.	0	0	9.56	0	0	0	0	0	0
IL-17A stimulated	412.9	217.217	613.245	464.3801	244.842	694.08	594.58	281.742	738.40
TSLP unnstimulated.	1.48	0	4.56	0.8765	0	4.1325	0	0	0
TSLP stimulated	2.73	0	5.63	1.8075	0	4.5102	0	0	1.47
IFN-γ unstimulated	0	0	1.29	0	0	0.6535	0	0	0.342
IFN-γ stimulated	7414.42	7062.79	7660.766	7286.909	6989.268	7692.97	14506.69	12989.9	15479.11
TNF-α unstimulated	3.23	0	8.08	1.67	0	3.812	0	0	3.469
TNF-α stimulated	5028.241	4088.149	7277.398	4673.764	3131.48	5822.979	6739.737	4037.38	8325.57
IL 23 unstimulated	0	0	119.7838	0	0	18.624	0	00	0
IL 23 stimulated	2065.771	1716.182	2474.541	1777.484	1374.51	2189.48	2740.232	2431.43	3165.234
Il 2 unstimulated	0	0	0	0	0	0	0	0	0
Il 2 stimulated	15270.85	11439.34	18045.51	12486.77	9462.354	15895.19	20461.73	14148.98	22306.8
Il 31 unstimulated	0	0	182.89	0	0	120.456	0	0	40.55475
Il 31 stimulated	99.973	50.535	218.85	77.881	27.62	246.869	60.818	49.55	80.9465
Il-6 unstimulated	0.145	0	4.69	1.1165	0	3.2325	0	0	2.0675
Il 6 stimulated	183.28	103.35	252.395	227.2875	130.5025	375.9222	93.578	51.40375	166.973

We report the median (50th percentile) and the interquartile range (25th, 75th percentile) as a measure of variability.

**Table 2B:** The mean and standard deviation of interleukins 17A, TSLP, IFN-γ, TNF-α, IL-2, IL-6, IL-23, and IL-31 (in picogram per milliliter) in patients with and without dupilumab therapy.

	The mean and standard deviation			Statistical analysis			
	Dupilumab yes 27 patients	Dupilumab no 62 Patients	Control group 34 subjects	KW test	DUP yes/DUP no	DUP no/control *p- value*	DUP yes/control *p- value*
IL 17A unnstimulated	12.184 s.d. 34.319	2.738 s.d. 8.622	0 0	0.00614			(p<0.01)
IL 17A stimulated	424.168 s.d. 235.916	518.172 s.d. 403.585	569.614 s.d. 411.451	0.32342			
TSLP unstimulated	6.251 s.d.13.592	9.078 s.d.21.565	1.3333 s.d.3.6288	0.00275		p<0.01	p<0.05
TSLP stimulated	6.472 s.d.12.898	9.571 s.d.20.404	1.604 s.d.3.476	0.00163		p<0.01	p<0.01
IFN-γ unstimulated	0.615 s.d.1.189	1.874 s.d.5.750	0.983 s.d.2.069	0.5472			
IFN-γ stimulated	7080.463 s.d.1754.862	7591.262 s.d.891.735	13504.03 s.d.2936.863	0.00000		p<0.001	p<0.001
TNF-α unstimulated	5.932 s.d.7.521	3.399 s.d.7.193	1.618 s.d.2.704	0.0069			p<0.01
TNF-α stimulated	5775.03 s.d.3211.729	5092.727 s.d.3319.893	6230.689 s.d.2963.677	0.0296		p<0.05	
IL 23 unstimulated	118.868 s.d 322.058	186.636 s.d 382.427	48.545 s.d 172.657	0.1656			
IL 23 stimulated	1831.412 s.d.750.190	2077.347 s.d 460.758	2556.128 s.d 920.351	0.00001		p<0.001	p<0.05
Il 2 unstimulated	0.0624 s.d 0.378	0.3811 s.d 1.980	0.1785 s.d 1.041	0.9834			
Il 2 stimulated	14411.66 s.d 9702.224	16877.12 s.d 9750.248	17902.72 s.d 7441.215	0.0055		p<0.01	
Il 31 unstimulated	184.028 s.d 409.248	162.231 s.d 341.178	74.0568 s.d 212.257	0.6590			
Il 31 stimulated	233.303 s.d 374.460	240.799 s.d 369.229	118.763 s.d 193.601	0.3684			
Il 6 unstimulated	6.2893 s.d 34.179	4.9850 s.d 11.923	3.6796 s.d 13.549	0.5123			
Il 6 stimulated	280.931 s.d 199.415	194.521 s.d 109.275	127.292 s.d 126.196	0.00003		p<0.001	p<0.05

The statistical comparison of the levels of interleukins between AD patients with and without dupilumabl therapy compared to control group.

We show the mean and standard deviation (s.d.) of interleukins (in picogram per milliliter).

The hypothesis of agreement was tested against the alternative that at least two groups differ from each other. Nonparametric Kruskal-Wallis analysis of variance with *post-hoc* Dunn’s test with Bonferroni modification of significance level was used (KW test).

We show a statistically significant difference when comparing the groups:

1) AD patients with dupilumab versus AD patients without dupilumab (DUP yes/DUP no).

2) AD patients without dupilumab versus control (DUP no/control).

3) AD patients with dupilumab versus control (DUP yes/control.

If the difference is statistically significant, we show *p - values*. If there is no space, there is no statistically significant difference.

Explanation: uns., unstimulated interleukins; s., stimulated interleukins; KW test, Kruskal-Wallis analysis; DUP, dupilumab.

In **Table 2B** we show the mean and standard deviation unstimulated and stimulated plazma levels of IL-17A, TSLP, IFN-γ, TNF-α, IL-2, IL-6, IL-23, and IL-31 in patients with and without dupilumab therapy and in control group. We show also the results of statistical comparison of interleukins between patients with and without dupilumab therapy compared to control group.

IL-17A: We recorded the significantly higher unstimulated levels of IL-17A in AD patients treated with dupilumab compared to control group (p<0.01). The difference in IL-17A stimulated in AD patients with and without dupilumab therapy compared to control group was not confirmed.

TSLP: Our analysis revealed significantly elevated TSLP levels in both AD patient groups regardless of dupilumab treatment compared to the control group, in both unstimulated and stimulated plasma samples (p < 0.01, p < 0.05).

IFN-γ: We recorded no significantly difference in unstimulated levels of IFN-γ in both AD patient groups-regardless of dupilumab treatment-compared to the control group. Our study confirmed significantly lower levels of stimulated IFN-γ in both untreated AD patients and those undergoing dupilumab therapy compared to healthy controls (p<0.001).

TNF-α: We recorded the significantly higher unstimulated levels of TNF-α in AD patients treated with dupilumab compared to control group (p<0.01) and significantly lower stimulated levels of TNF-α in AD patients without dupilumab compared to control group (p<0.05).

IL- 23: We recorded the significantly lower stimulated levels of IL-23 in both AD patient groups-regardless of dupilumab treatment-compared to the control group, (p < 0.001, p < 0.05). The difference in IL-23 unstimulated in AD patients with and without dupilumab therapy compared to control group was not confirmed.

IL-2: We recorded the significantly lower stimulated levels of IL-2 in AD patients treated with dupilumab compared to control group (p<0.01). The difference in IL-2 unstimulated in AD patients with and without dupilumab therapy compared to control group was not confirmed.

IL-31: We recorded no difference in stimulated and unstimulated levels of IL-31 in both AD patient groups-regardless of dupilumab treatment-compared to the control group,

IL-6: We recorded the significantly higher stimulated levels of IL-6 in both AD patient groups-regardless of dupilumab treatment-compared to the control group, (p < 0.001, p < 0.05). The difference in IL-6 unstimulated in AD patients with and without dupilumab therapy compared to control group was not confirmed.

In figures to **Table 2A** we show the significant differences in levels of examined cytokines in AD patients and in control group.

**Figure 1:**
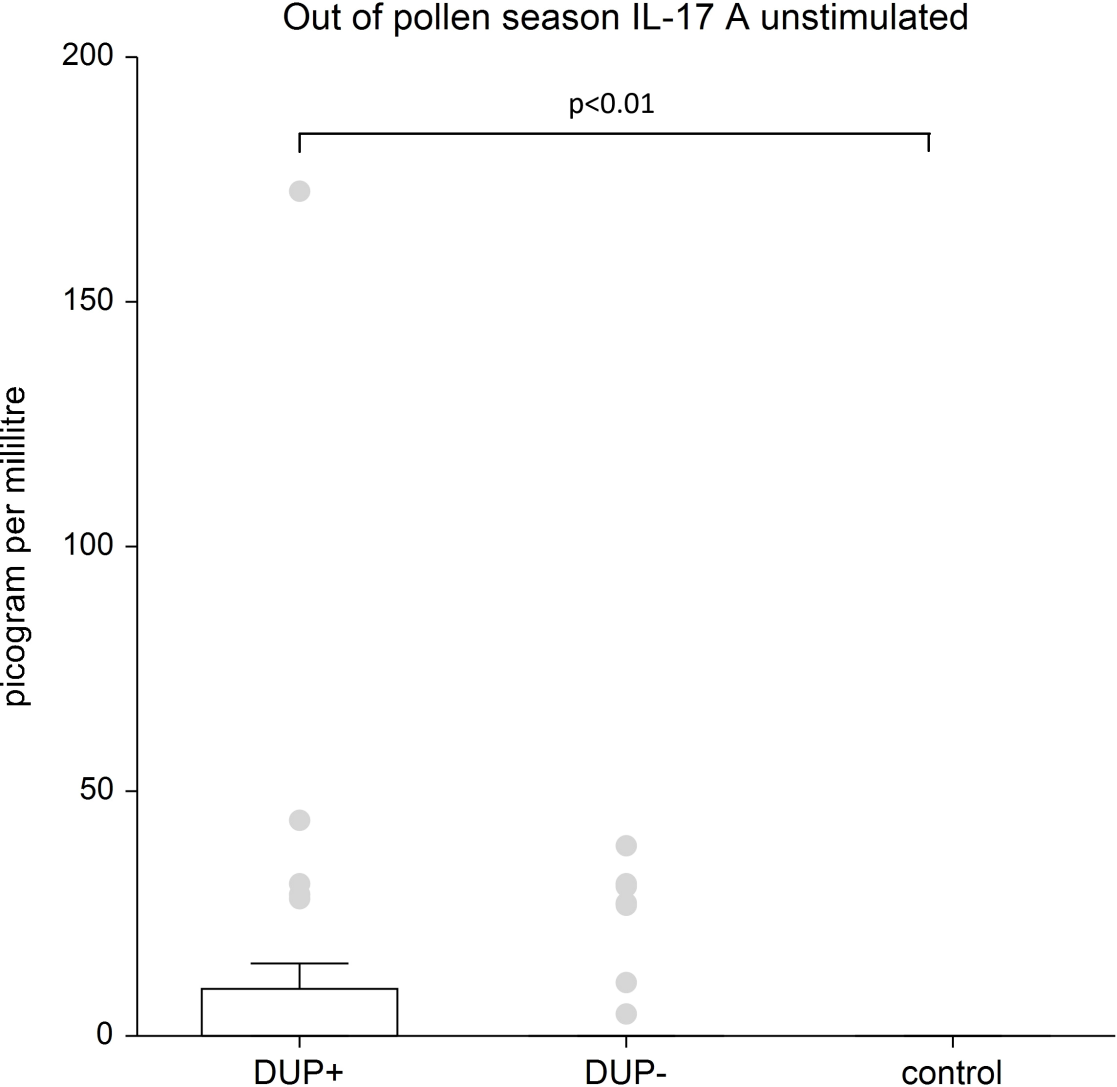
Shows unstimulated plasma samples of IL 17A. The unstimulated plasma samples of IL 17A is significantly higher in AD patients with dupilumab compared to control group (p=0.00614). There is no significant difference between AD patients without dupilumab to control group. Box whisker plot of median-the top edge of the box is the 75th percentile, median=0, dots are outliers.

**Figure 1** shows unstimulated plasma samples of IL 17A. The unstimulated plasma samples of IL 17A is significantly higher in AD patients with dupilumab compared to control group (p<0.01).

**Figure 2:**
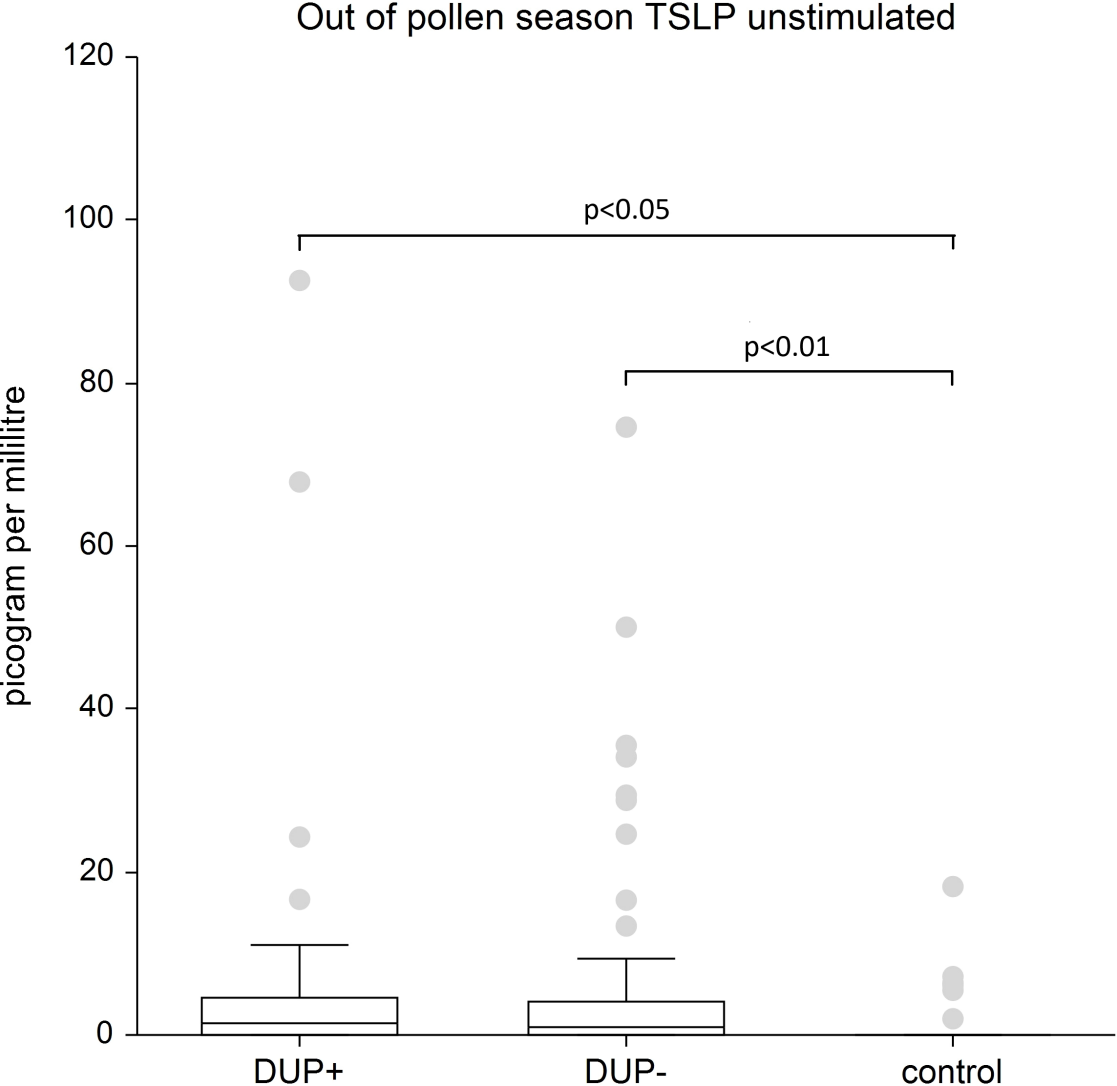
Shows unstimulated plasma samples of TSLP. The unstimulated plasma samples of TSLP is significantly higher in AD patients both with dupilumab therapy (p<0.05) and in AD patients without any systemic therapy compared to control group (p<0.01). The box is the 25th and 75th percentile, the line in the box is the median (50th percentile), dots are outliers.

**Figure 2** shows unstimulated plasma samples of TSLP. The unstimulated plasma samples of TSLP is significantly higher in AD patients both with dupilumab therapy (p<0.05) and in AD patients without any systemic therapy compared to control group (p<0.01).

**Figure 3:**
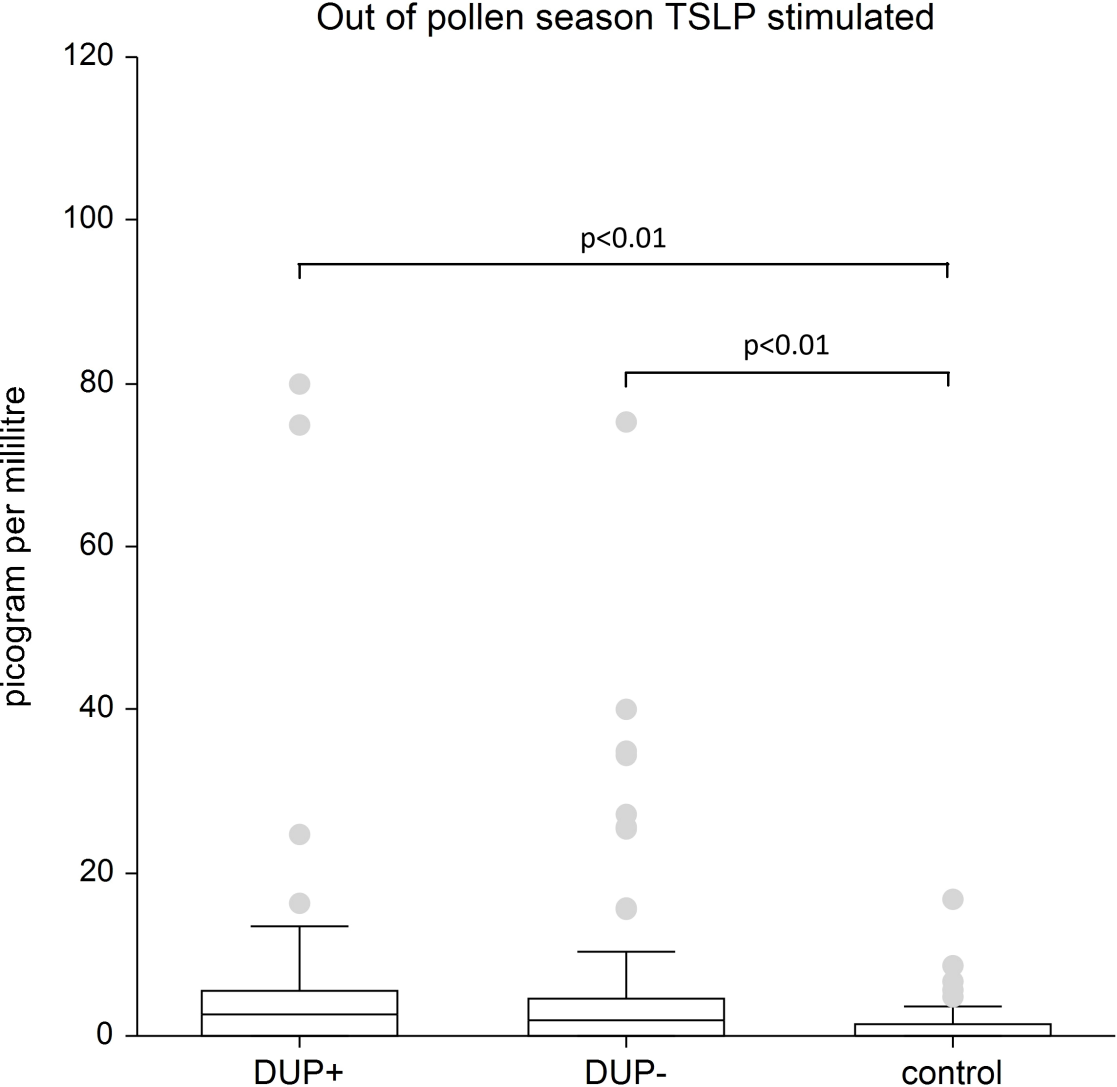
Shows stimulated plasma samples of TSLP. The stimulated plasma samples of TSLP is significantly higher in AD patients both with dupilumab therapy and in AD patients without any systemic therapy compared to control group (p<0.01). The box is the 25th and 75th percentile, the line in the box is the median (50th percentile), dots are outliers.

**Figure 3** shows stimulated plasma samples of TSLP. The stimulated plasma samples of TSLP is significantly higher in AD patients both with dupilumab therapy and in AD patients without any systemic therapy compared to control group (p<0.01).

**Figure 4:**
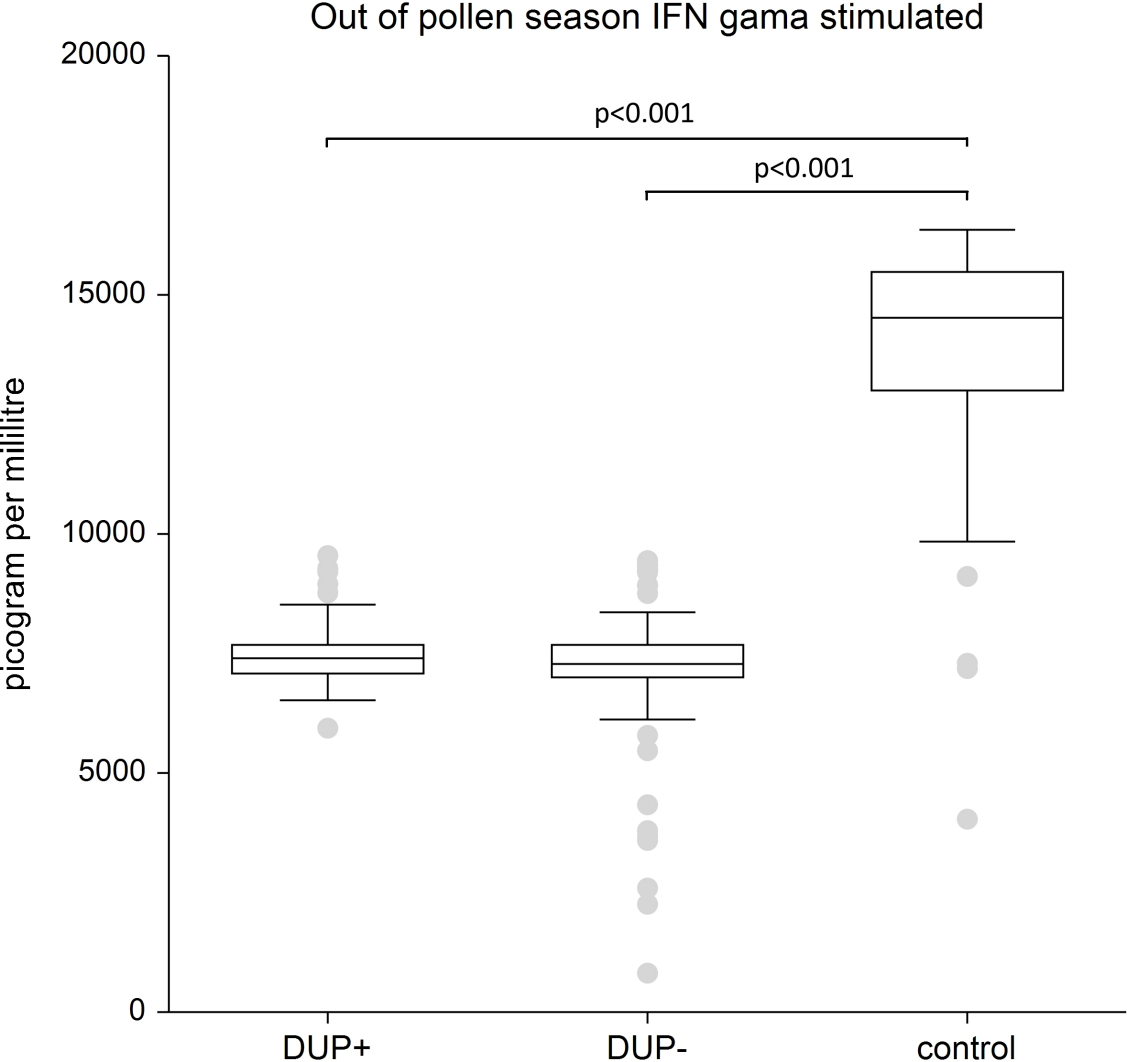
Shows stimulated plasma samples of interferon gama (IFN gama). The stimulated plasma samples of IFN gama is significantly lower in AD patients both with dupilumab therapy and in AD patients without any systemic therapy compared to control group (p <0.001). The box is the 25th and 75th percentile, the line in the box is the median (50th percentile), dots are outliers.

**Figure 4** shows stimulated plasma samples of IFN-γ. The stimulated plasma samples of IFN-γ is significantly lower in AD patients both with dupilumab therapy and in AD patients without any systemic therapy compared to control group (p<0.001).

**Figure 5:**
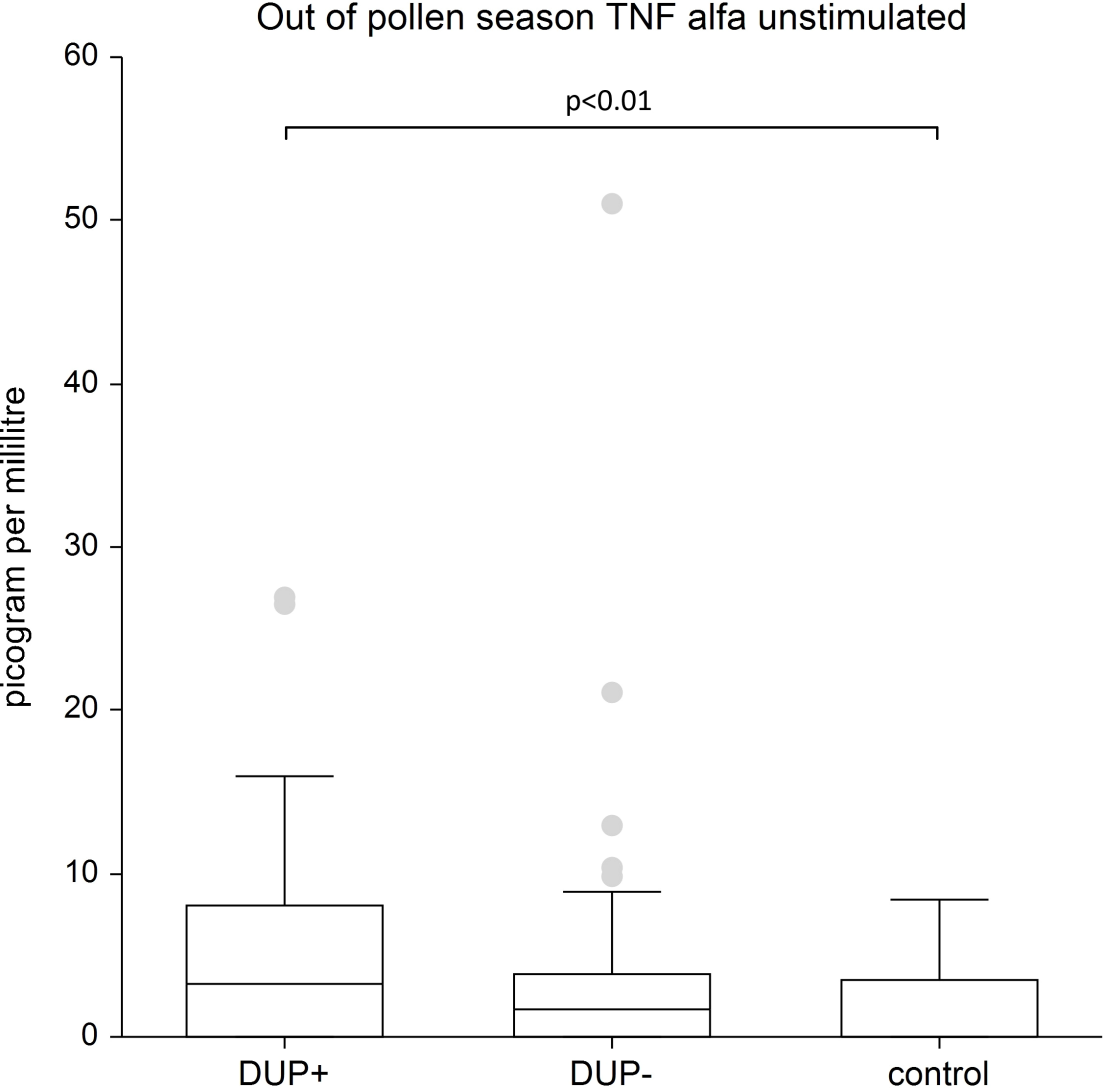
Shows unstimulated plasma samples of tumor necrosis alfa (TNF alfa). The stimulated plasma samples of TNF alfa is significantly higher in AD patients treated with dupilumab compared to control group (p<0.01). Box whisker plot of median - the top edge of the box is the 75th percentile, the line in the box is the median (50th percentile), 25th percentile = 0, dots are outliers.

**Figure 5** shows unstimulated plasma samples of TNF-α. The stimulated plasma samples of TNF-α is significantly higher in AD patients treated with dupilumab compared to control group (p<0.01).

**Figure 6:**
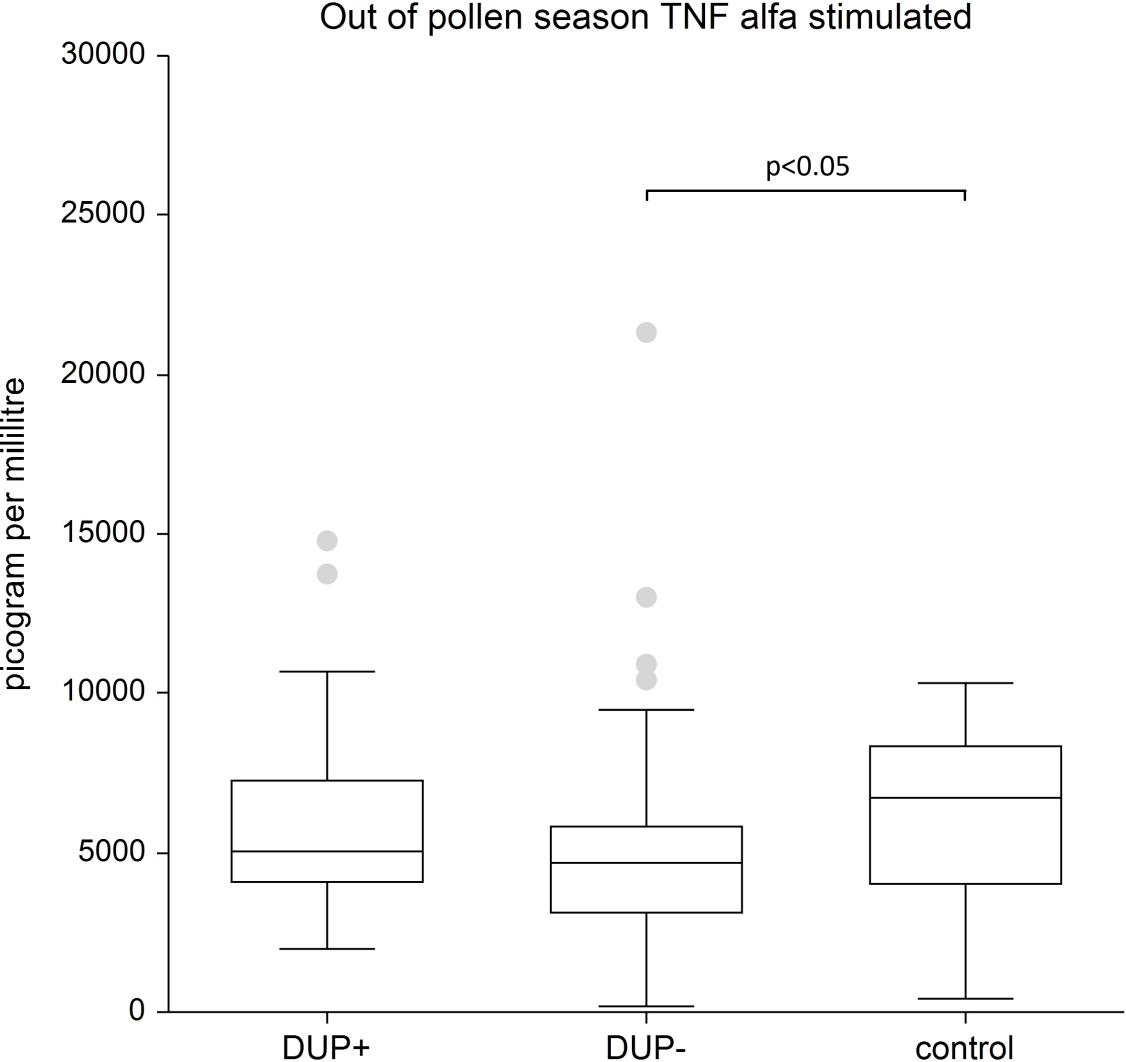
Shows stimulated plasma samples of tumor necrosis alfa (TNF alfa). The stimulated plasma samples of TNF alfa is significantly lower in AD patients without any systemic therapy compared to control group (p<0.05). The box is the 25th and 75th percentile, the line in the box is the median (50th percentile), dots are outliers.

**Figure 6** shows stimulated plasma samples of TNF-α. The stimulated plasma samples of TNF-α is significantly lower in AD patients without any systemic therapy compared to control group (p<0.05).

**Figure 7:**
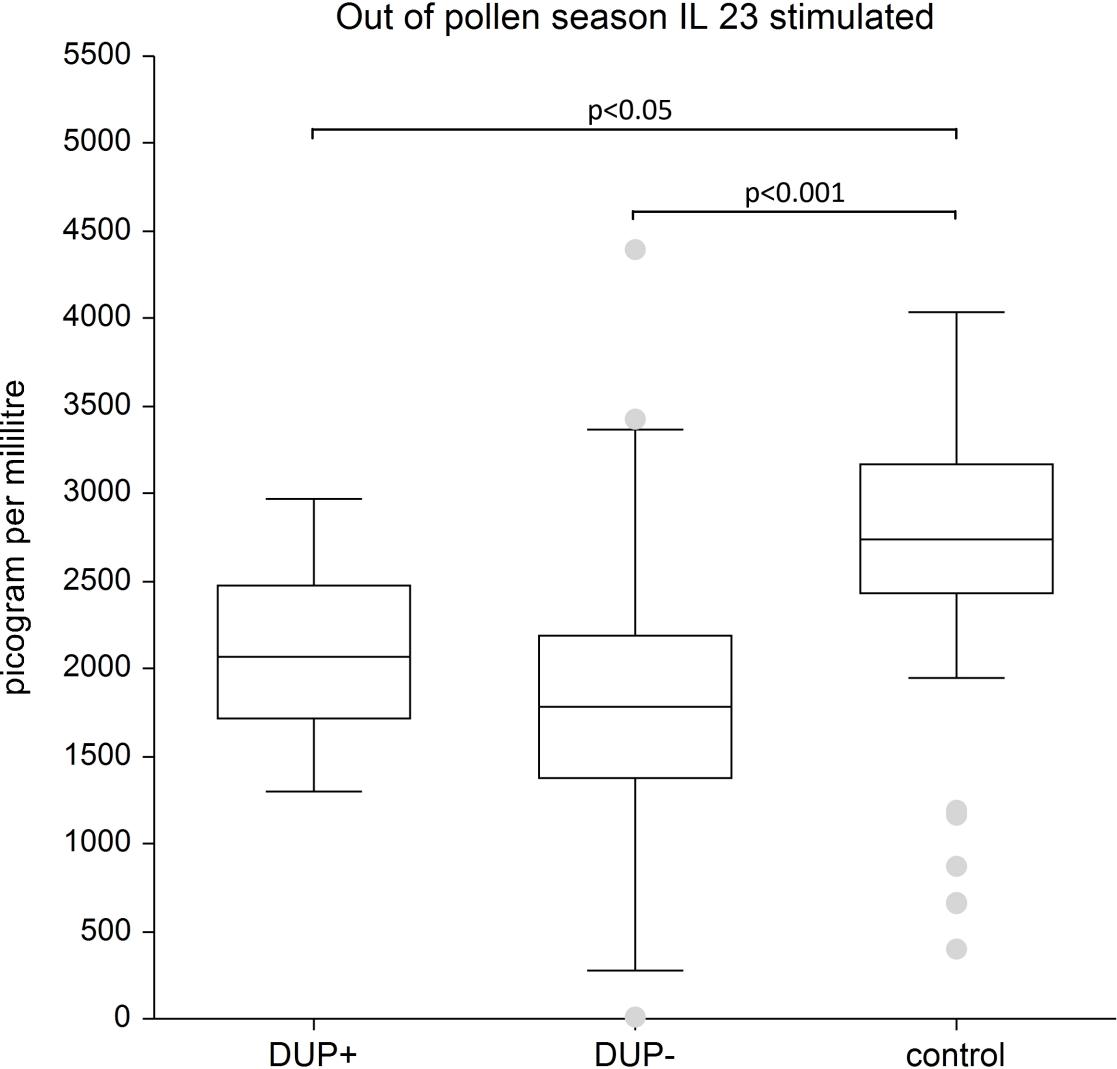
Shows stimulated plasma samples of IL-23. We show lower level of stimulated IL-23 in AD patients with and without dupilumab therapy compared to healthy subjects. The box is the 25th and 75th percentile, the line in the box is the median (50th percentile), dots are outliers.

**Figure 7** displays lower level of stimulated IL- 23 in AD patients with and without dupilumab therapy compared to healthy subjects.

**Figure 8:**
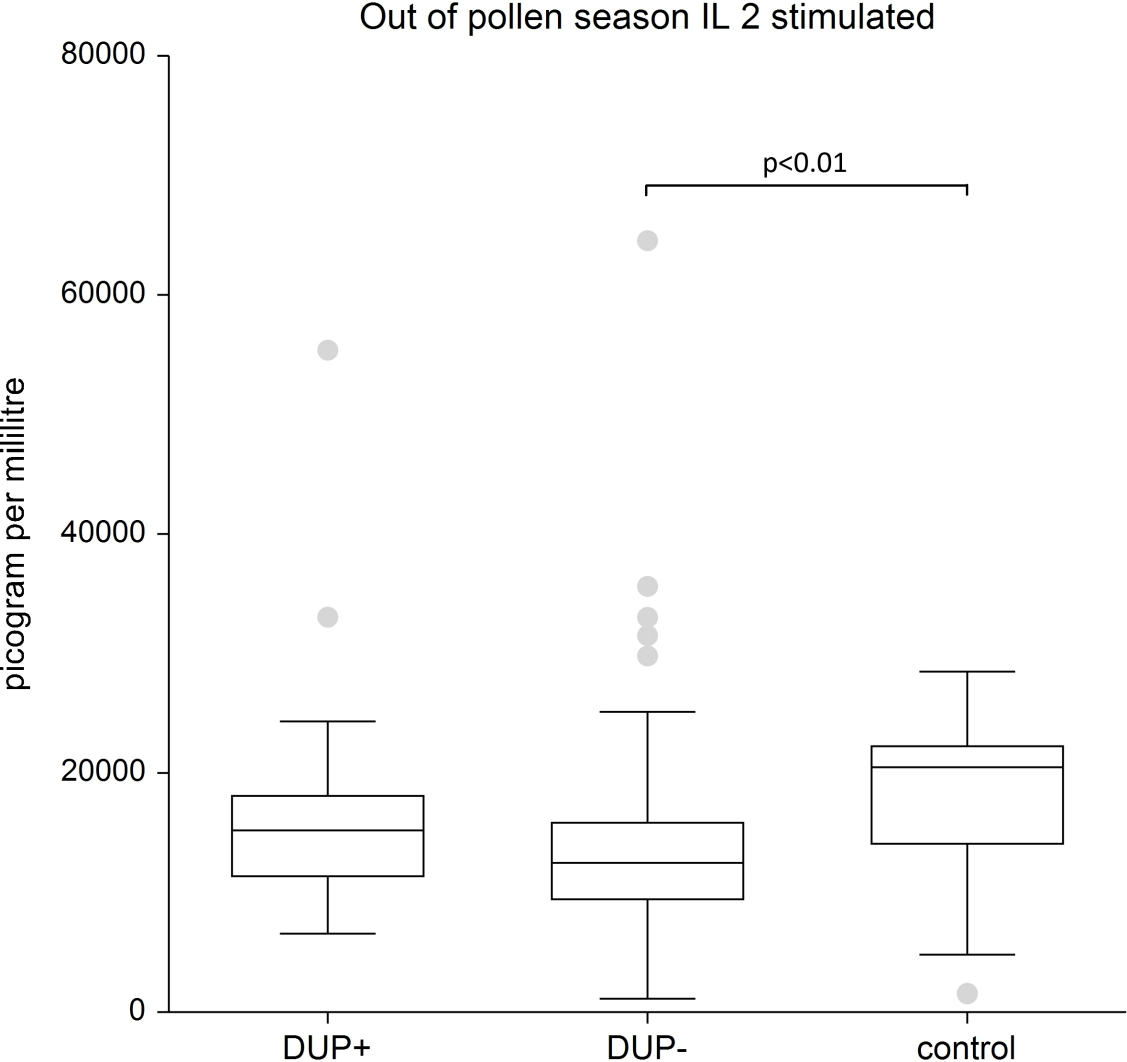
Shows stimulated plasma samples of IL-2. We show lower level of stimulated IL 2 in AD patients without dupilumab therapy compared to healthy subjects. The box is the 25th and 75th percentile, the line in the box is the median (50th percentile), dots are outliers.

**Figure 8** shows lower level of stimulated IL- 2 in AD patients without dupilumab therapy compared to healthy subjects.

**Figure 9:**
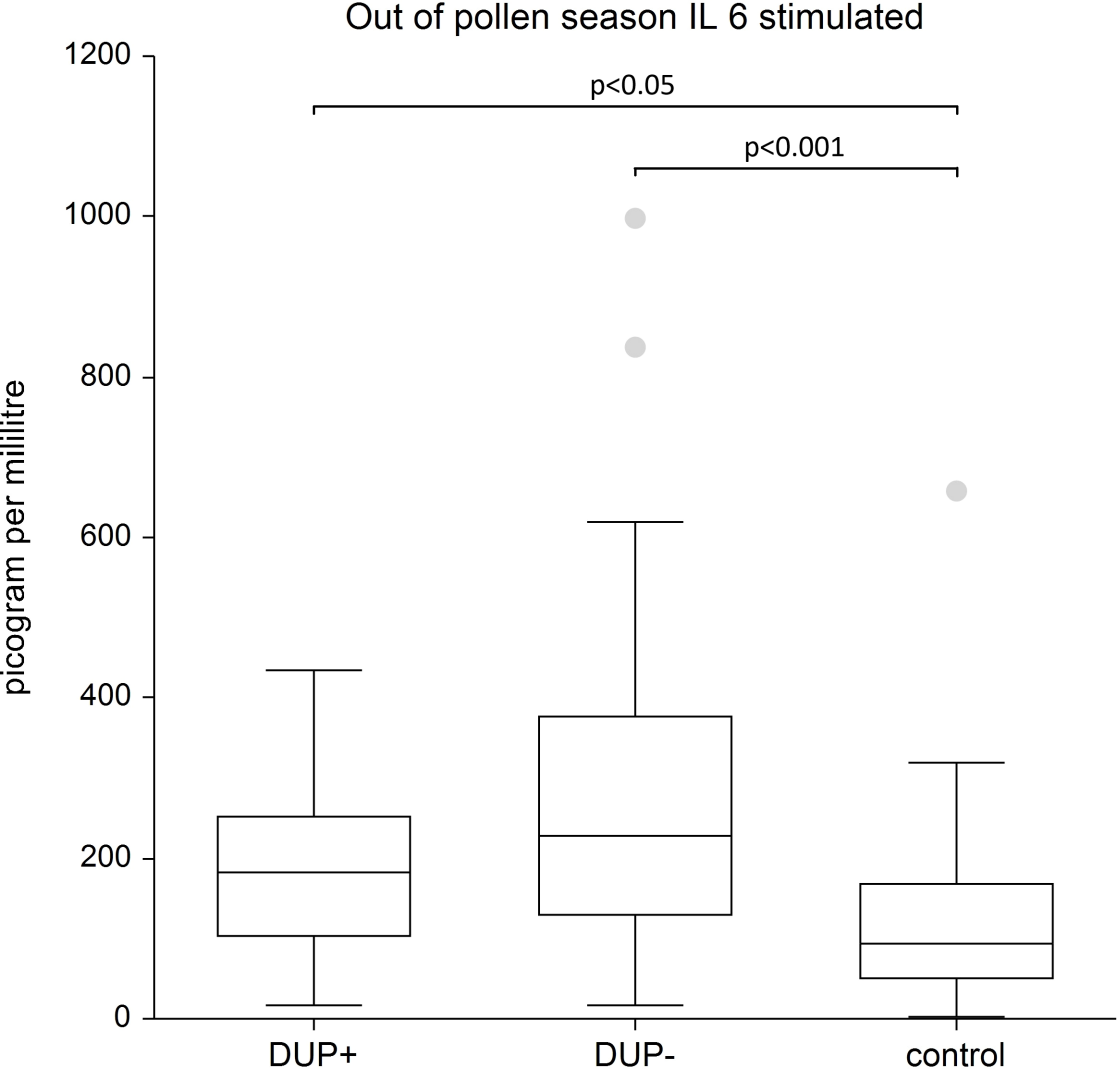
Shows stimulated plasma samples of IL-6. We show higher level of stimulated IL - 6 in AD patients with and without dupilumab therapy compared to healthy subjects. The box is the 25th and 75th percentile, the line in the box is the median (50th percentile), dots are outliers.

**Figure 9** shows higher level of stimulated IL- 6 in AD patients with and without dupilumab therapy compared to healthy subjects.

## Discussion

Our study provides novel insights into the systemic immune profile of AD patients, particularly those receiving dupilumab therapy, a monoclonal antibody targeting the IL-4 receptor α (IL-4Rα). The findings highlight persistent immune activation and altered cytokine responsiveness under biologic treatment, underscoring the complexity of immune regulation in AD.

At first, we discuss the relevance of immune cell stimulation. Accurate assessment of plasma cytokine levels-including IL-17A, TSLP, IFN-γ, TNF-α, IL-2, IL-6, IL-23, and IL-31-requires immune cell stimulation to reveal functional cytokine-producing capacity. In this study, blood samples were analyzed under both unstimulated conditions and following stimulation with phorbol myristate acetate (PMA) and ionomycin. This protocol induces robust, non-specific activation of both innate and adaptive immune cells, thereby markedly enhancing cytokine secretion. Under resting conditions, circulating cytokines are often present at very low or undetectable concentrations, limiting the assessment of immune competence. Stimulation allows evaluation of cellular responsiveness and standardizes immune activation across samples, reducing interindividual variability and increasing assay sensitivity, particularly in multiplex platforms such as Human Cytokine Luminex. Furthermore, this approach enables the detection of dysregulated or exhausted immune responses, providing mechanistic insight into both disease pathology and treatment effects. Overall, immune stimulation represents a powerful strategy for uncovering the dynamic functional potential of immune cells beyond baseline activity (41, 42).

Our study demonstrates Th17/TNF-α pathway activation during dupilumab therapy. We observed significantly elevated unstimulated plasma levels of IL-17A and TNF-α in dupilumab-treated patients compared with healthy controls, whereas stimulated cytokine levels were comparable across groups. In parallel, TSLP levels remained persistently elevated in both treated and untreated AD patients, while IFN-γ levels remained uniformly low regardless of therapy. These findings reflect complex immune modulation during IL-4/IL-13 blockade. While dupilumab effectively suppresses Th2-driven inflammation, our data suggest that inhibition of this dominant pathway may unmask or enhance alternative immune axes, particularly Th17- and TNF-α–mediated responses. This observation is consistent with reports of psoriasiform eruptions in patients receiving dupilumab, which are thought to reflect a shift toward Th1/Th17 immune dominance following Th2 suppression (9, 10). Elevated unstimulated IL-17A levels suggest a baseline immune deviation, potentially driven by immune plasticity or the presence of AD endotypes characterized by mixed or Th17-skewed inflammation, such as intrinsic AD or chronic lesions.

Blockade of IL-4Rα inhibits both IL-4 and IL-13 signaling, key drivers of Th2 polarization. This interruption may relieve inhibitory signals on Th17 pathways, allowing increased IL-17A production in the baseline immune milieu. Such compensatory immune reprogramming has been described in studies investigating immune plasticity, wherein suppression of one axis results in the emergence of alternative inflammatory pathways (9). Psoriasiform erythema has been reported in children with AD undergoing dupilumab therapy (10), and it has been proposed that suppression of dominant Th2 responses may promote Th1/Th17 predominance, culminating in psoriasis-like disease. In this context, baricitinib has emerged as a potential therapeutic option with significant efficacy in managing psoriasiform eruptions associated with dupilumab therapy (11).

In our study we confirmed persistent TSLP elevation despite Th2 blockade; our findings further underscore the persistent role of TSLP in AD pathogenesis, even in the setting of IL-4/IL-13 inhibition. Acting upstream in the inflammatory cascade, TSLP promotes dendritic cell activation and Th2 differentiation. Its continued elevation in both treated and untreated patients likely reflects ongoing epithelial stress or subclinical inflammation and reinforces its relevance as a biomarker and potential therapeutic target in AD (12–24). Longitudinal studies are warranted to clarify TSLP behavior over the course of dupilumab treatment and to evaluate whether combined targeting of TSLP and Th2 pathways could improve clinical outcomes.

Regarding IFN-γ signaling in AD, we confirmed persistently low levels of IFN-γ in both AD patients without any systemic treatment and those receiving dupilumab. This observation aligns with the established immunopathology of AD, in which Th2 cytokines such as IL-4 and IL-13 suppress Th1 differentiation and IFN-γ production (25–29). IL-4 inhibits IFN-γ gene transcription and downregulates IL-12 receptor expression on T cells, while IL-13 interferes with STAT1 signaling in keratinocytes, collectively impairing Th1 responses. These immunological insights have prompted clinical trials investigating recombinant IFN-γ in patients with severe, treatment-refractory AD. Such therapy was proposed to correct immune imbalances by reducing serum IgE and IL-4 levels while restoring immune equilibrium, leading to clinical improvement (25). Expert opinion suggests that recombinant IFN-γ may particularly benefit pediatric AD populations and patients prone to recurrent skin infections. However, further studies are necessary to clarify its impact on infection susceptibility in AD (26).

Results of our study point to TNF-α dynamics and immune exhaustion. Unstimulated TNF-α levels were significantly elevated in AD patients compared with healthy controls, with the highest median concentrations observed in dupilumab-treated individuals. This suggests that residual inflammatory activity persists despite effective Th2 suppression and may be mediated by TNF-α–driven pathways. Elevated baseline TNF-α in treated patients may reflect compensatory immune mechanisms or TNFR2-mediated regulatory processes, which support Treg function and immune tolerance. In contrast, stimulated TNF-α responses were highest in healthy controls, reflecting preserved immune responsiveness. AD patients—particularly those not receiving dupilumab—exhibited significantly diminished TNF-α production upon stimulation, consistent with chronic immune activation and cytokine exhaustion (27, 30–32). Dupilumab-treated patients demonstrated intermediate TNF-α responses, suggesting partial restoration of immune responsiveness following therapy (30). These findings align with previous reports documenting increased expression of TNFR1 and TNFR2 on immune cells in AD, correlating with disease severity (27, 30–32). Moreover, metabolomic studies reporting reductions in serum TNF-α after dupilumab treatment support the notion of indirect TNF-α modulation via broader immune rebalancing (27).

Other results point to IL-23 dysregulation and immune priming. IL-23 levels following stimulation were significantly lower in AD patients than in healthy controls, irrespective of dupilumab treatment. Conversely, unstimulated IL-23 levels were higher in AD patients, although this difference did not reach statistical significance. These findings suggest that immune cells in AD exist in a pre-activated or “primed” state, producing IL-23 constitutively as part of chronic low-grade inflammation. The blunted IL-23 response upon stimulation may indicate immune exhaustion or negative feedback mechanisms limiting further cytokine induction. Alternatively, regulatory pathways—such as enhanced IL-10 signaling or increased Treg activity—may suppress IL-23 production following stimulation, particularly in the context of dupilumab-induced immune rebalancing. Yamamura et al. emphasize the variable role of IL-23 in AD subtypes and propose that IL-23 expression reflects immune priming and regulatory feedback in chronic disease, consistent with our findings (43). Similarly, Fania et al. describe IL-23 as a biomarker of Th17 activity influenced by regulatory cytokines and Treg dynamics (44).

We confirmed IL-2 deficiency and Treg dysfunction, because we identified significantly lower stimulated IL-2 levels in AD patients not receiving dupilumab compared with healthy controls. IL-2 is essential for T-cell proliferation and maintenance of regulatory T cells, and its deficiency may reflect impaired immune regulation and T-cell exhaustion in untreated AD. This observation is consistent with previous reports linking reduced IL-2 availability to compromised Treg function and chronic inflammation (45).

Notably, dupilumab-treated patients demonstrated a trend toward normalization of IL-2 levels, suggesting that Th2 blockade may indirectly restore immune homeostasis. These findings carry therapeutic implications, as strategies enhancing IL-2 signaling or Treg activity may complement existing biologic therapies. Heikinheimo also reported reduced IL-2 levels in AD patients and linked this deficit to impaired regulatory function, supporting our observation of IL-2 normalization following dupilumab therapy (46).

Results of our study show persistent IL-6 elevation and innate immune activation. IL-6 levels were elevated in AD patients compared with healthy controls, regardless of dupilumab treatment. IL-6 is a pleiotropic cytokine produced by keratinocytes, fibroblasts, and immune cells in response to barrier disruption and microbial exposure (47). Its persistent elevation indicates that IL-6–driven inflammation is not sufficiently addressed by Th2-targeted therapies alone. This observation supports the concept that AD pathogenesis involves multiple immune axes, including innate immunity and possibly Th1/Th17 pathways, particularly in chronic disease stages. Persistent IL-6 elevation may also contribute to systemic inflammation and associated comorbidities, such as asthma and cardiovascular risk, emphasizing the need for broader immunomodulatory strategies. Both Fania et al. and Yamamura et al. identify IL-6 as a key mediator of barrier dysfunction and innate immune activation in AD, noting its persistence despite Th2-directed treatment (43, 55).

Finally, IL-31 has been identified as a central mediator of AD-associated pruritus and neuroimmune signaling. Yamamura et al. describe persistent IL-31 activity as a potential explanation for residual itch in dupilumab-treated patients, highlighting the importance of IL-31–targeted therapies for comprehensive symptom control (43).

The interpretation of our findings is limited by the inability to clearly distinguish dupilumab-induced immune changes from pre-existing immune heterogeneity in atopic dermatitis. Due to the cross-sectional design and lack of baseline cytokine data, both treatment effects and inherent immune endotypes may contribute to the observed cytokine patterns. Atopic dermatitis is a heterogeneous disease, and patients with non-Th2-dominant immune profiles may be predisposed to atypical or paradoxical responses under Th2-targeted therapy (43, 44). However, the persistence of elevated IL-17A and TNF-α in dupilumab-treated patients despite marked clinical improvement suggests immune rebalancing rather than inflammation driven by disease severity alone. Additionally, sustained elevation of TSLP and IL-6 in both treated and untreated patients indicates ongoing epithelial and innate immune activation not fully resolved by Th2 blockade. Together, these findings support the concept that dupilumab suppresses Th2 signaling while permitting continued activity of alternative inflammatory pathways, including Th17 and innate immune axes.

The observed cytokine patterns also raise the possibility that certain circulating cytokines may serve as biomarkers of treatment response or immune phenotype stratification. Persistent elevation of IL-17A or TNF-α despite clinical improvement could identify patients with mixed or non-Th2 immune endotypes who may be prone to residual symptoms, partial response, or paradoxical inflammatory reactions during dupilumab therapy. Similarly, sustained elevation of TSLP and IL-6 may reflect ongoing epithelial stress and innate immune activation, highlighting pathways not fully addressed by IL-4/IL-13 blockade.

Although no single cytokine clearly distinguished dupilumab-treated from untreated patients in this study, composite cytokine profiles or longitudinal changes within individuals may be more informative than absolute cross-sectional differences. Future prospective studies evaluating cytokine trajectories before and after dupilumab initiation, and correlating them with clinical outcomes, could clarify whether systemic markers such as IL-17A, IL-6, or TSLP have predictive value for treatment response, durability, or adverse immune shifts.

Although differences were noted between AD patients treated with dupilumab and healthy controls, the absence of differences in cytokine levels in dupilumab versus non-dupilumab support the fact that dupi-treated patients had a milder course, which does not correlate with the absence of differences in cytokine levels.

Our findings underscore the complexity of AD immunology and the limitations of therapies targeting a single pathway. While dupilumab effectively reduces Th2-mediated inflammation, residual activity of cytokines like IL-6 and persistent suppression of IL-23 indicate that additional targets may be necessary for complete disease control. Emerging treatments such as JAK inhibitors, which modulate multiple cytokine signals, or agents targeting IL-22 and IL-31, may offer complementary benefits (47, 48). Furthermore, monitoring cytokine profiles could help stratify patients and guide personalized therapy.

Recent real-world studies, scoping reviews, and mechanistic reports published in 2023–2025 describe dupilumab-associated immune skewing toward Th17 and Th1 pathways. These newer publications introduce the concept of biologic-induced “immune drift” and document *de novo* IL-17A expression and psoriasiform reactions under IL-4/IL-13 blockade. Study of Su Z et al. supports psoriasiform reactions Th17Th1 imbalance after IL4/IL13 blockade (49). Camela et al. demonstrates immune skewing outside clinical třísla and supports real - world relevance (50).

## Limitations

One major limitation of this study is the difference in disease severity between comparison groups at the time of sampling. Patients on dupilumab had achieved substantial improvement, with most presenting mild AD, whereas the untreated group included individuals with moderate-to-severe disease. This imbalance is a significant confounder, as disease activity itself can influence cytokine profiles. Although dupilumab-treated patients previously had severe AD, their current immune status likely reflects both therapeutic effects and reduced inflammation. A longitudinal design, tracking cytokine levels in the same patients before and after dupilumab initiation, would better isolate treatment effects and provide more robust causal insights.

Another limitation is the cross-sectional design. Cross-sectional studies are observational studies that analyze data from a population at a single point in time. They are often used to measure the prevalence of health outcomes, to understand the determinants of health, and to describe population characteristics. Unlike other types of observational studies, cross-sectional studies do not follow individuals over time. Our study is for obtaining preliminary evidence when planning a future advanced study (51).

## Conclusion

Our study highlights the immune complexity of AD and the multifaceted effects of dupilumab beyond Th2 suppression. The observed cytokine patterns suggest that immune rebalancing during treatment may lead to activation of alternative pathways, such as Th17 and TNF-α, which could influence clinical outcomes and adverse effects. These findings support the need for personalized approaches in AD management and encourage further research into combination therapies targeting upstream mediators like TSLP or downstream cytokines such as IL-17A and TNF-α. Elevated baseline IL-23 levels in AD patients suggest a primed immune state, while the blunted IL-23 response upon stimulation indicates possible immune exhaustion or regulatory suppression. Reduced IL-2 production in untreated patients points to impaired Treg function, which may be partially restored by Th2 blockade with dupilumab. Persistent elevation of IL-6 and activity of IL-31, despite dupilumab treatment, underscores the involvement of multiple immune pathways beyond Th2, including innate and neuroimmune mechanisms. These findings emphasize that targeting a single pathway is insufficient for complete disease control. Future strategies should consider broader immunomodulation—such as JAK inhibitors or agents targeting IL-22 and IL-31—and cytokine profiling to guide personalized therapy.

## Data Availability

The raw data supporting the conclusions of this article will be made available by the authors, without undue reservation.
